# Green and Economic Fabrication of Zinc Oxide (ZnO) Nanorods as a Broadband UV Blocker and Antimicrobial Agent

**DOI:** 10.3390/nano10030530

**Published:** 2020-03-15

**Authors:** Seyedeh-Masoumeh Taghizadeh, Neha Lal, Alireza Ebrahiminezhad, Fatemeh Moeini, Mostafa Seifan, Younes Ghasemi, Aydin Berenjian

**Affiliations:** 1 Department of Pharmaceutical Biotechnology, School of Pharmacy, and Pharmaceutical Sciences Research Center, Shiraz University of Medical Sciences, 71348-14336 Shiraz, Iran; taghizm@sums.ac.ir (S.-M.T.); fmoeini@yahoo.com (F.M.); ghasemiy@sums.ac.ir (Y.G.); 2 School of Engineering, Faculty of Science and Engineering, The University of Waikato, Hamilton 3240, New Zealand; neha.natasha.lal@gmail.com (N.L.); mostafa.seifan@waikato.ac.nz (M.S.); 3 Department of Medical Nanotechnology, School of Advanced Medical Sciences and Technologies, Shiraz University of Medical Sciences, 71348-14336 Shiraz, Iran

**Keywords:** controlled synthesis, zinc oxide nanorods, secretory compounds, *Chlorella vulgaris*, broadband UV blocker, antimicrobial activity

## Abstract

Zinc oxide (ZnO) nanoparticles have gained widespread interest due to their unique properties, making them suitable for a range of applications. Several methods for their production are available, and of these, controlled synthesis techniques are particularly favourable. Large-scale culturing of *Chlorella vulgaris* produces secretory carbohydrates as a waste product, which have been shown to play an important role in directing the particle size and morphology of nanoparticles. In this investigation, ZnO nanorods were produced through a controlled synthesis approach using secretory carbohydrates from *C. vulgaris*, which presents a cost-effective and sustainable alternative to the existing techniques. Fourier transform infrared (FTIR) spectroscopy, X-ray powder diffraction (XRD) analysis, transmission electron microscopy (TEM), and UV-Vis spectroscopy were used to characterise the nanorods. The prepared nanorods exhibited a broad range of UV absorption, which suggests that the particles are a promising broadband sun blocker and are likely to be effective for the fabrication of sunscreens with protection against both UVB (290–320 nm) and UVA (320–400 nm) radiations. The antimicrobial activity of the prepared nanorods against Gram-positive and Gram-negative bacteria was also assessed. The nanostructures had a crystalline structure and rod-like appearance, with an average length and width of 150 nm and 21 nm, respectively. The nanorods also demonstrated notable antibacterial activity, and 250 μg/mL was determined to be the most effective concentration. The antibacterial properties of the ZnO nanorods suggest its suitability for a range of antimicrobial uses, such as in the food industry and for various biomedical applications.

## 1. Introduction

Nanoparticles have widespread applications in various fields such as science, technology, and medicine, due to their unique physicochemical and biological properties. Zinc oxide (ZnO) nanoparticles, in particular, have gained considerable interest due to their generally recognised as safe (GRAS) status, large bandwidth, high exciton binding energy, and novel surface chemical properties [1,2,3,4]. These features make them suitable for a vast range of industrial and other applications, such as in the field of electronics, optics, and biomedicine, as well as in the food, cosmetic, and chemical industries [1,2,3,4]. The nanoscale size of ZnO particles provides a large surface-area-to-volume ratio and confers unique chemical, biological, mechanical, electrical, structural, morphological, and optical properties that are not observed in the bulk material, and these play a fundamental role in their suitability for an assortment of novel applications [3].

As a result of the high demand for ZnO nanoparticles, several methods have been developed for their production, and variation in the prominent chemical and physical parameters such as the pH, solvent type, precursors, and temperature enables the production of nanoparticles with different morphologies and properties that can be tailored to specific applications [3]. The shape, size, and morphology of ZnO nanostructures are largely attributable to the method of production and the presence of controlling agents that are used during the synthesis procedure. To date, ZnO nanoparticles have been successfully fabricated in a vast range of shapes and sizes such as microstars [5], microrods [6], micro-octahedrons [7], microflowers [8], nanowires [9], nanorods [2,10,11,12,13,14], nanosheets [15,16], nanobelts [17,18], nanorings, nanosprings, nanocombs, nanosaws, and nanopropellers [15]. Different methods have been frequently used for the synthesis of such nanoparticles, and these include the sol–gel technique [11,19], microemulsion synthesis [20], mechanochemical processing [21], sonochemical synthesis, microwave-assisted synthesis [14,22,23], vapour transport process [24], spray pyrolysis, spray drying [9], supercritical water processing [25], thermal decomposition of organic precursors [26,27], radio frequency (RF) plasma synthesis [28], direct and homogenous precipitation [7,29], self-assembling [30], hydrothermal processing [8,31], and precipitation [5,32]. 

Precipitation synthesis is one of the facile approaches in this regard that is at a new stage of development, where it is a common requirement to control the properties of the obtained nanostructures. A diverse range of compounds have been implemented to control the synthesis reactions. These compounds drive the crystal growth pattern and enable the production of ZnO nanostructures with unique shapes. The suitability of surface-active agents [33], polymers [34], and carbohydrates [35] to control the ZnO synthesis reaction has been widely investigated. It has been shown that, by employing increasing concentrations of polyethylene glycol (PEG), the transformation into rod-shaped structures can be achieved [2,34]. Controlling agents can also be obtained from microbial sources. Microorganisms have been vastly employed for the synthesis of nanoparticles, the majority of which are bacteria and fungi; however, the use of such microorganisms has several drawbacks [1]. The primary disadvantages are the requirement for careful monitoring to avoid contamination, the high cost of the growth media, the potential for the microorganisms to have an adverse effect on human health, and the need to screen suitable microbes, which tends to be a time-consuming process [1,36]. Consequently, the use of algae for the synthesis of nanoparticles is a favourable alternative in the microbial realm, as it offers several advantages and is a more sustainable option [36]. Algae have simple energy and nutrient requirements, because they use sunlight as an energy source, carbon dioxide as a carbon source, and ammonium salts as a nitrogen source; therefore, they present a highly economic alternative for large-scale processes [36].

To date, algae have been employed for the synthesis of silver and gold nanoparticles, but their potential for the production of ZnO nanoparticles has not yet been investigated [1]. Among microalgae, *C. vulgaris* is one of the most favourable species for the production of ZnO nanoparticles. Recently, the large-scale culturing of *C. vulgaris* for the production of biomass has gained considerable interest [36]. This process produces large quantities of culture supernatant as a waste stream, which results in environmental pollution [36]. Carbohydrates are the primary secretory bioactive compound present in the culture supernatant that can be employed in the synthesis of ZnO nanoparticles [37]. These secretory carbohydrates play a key role in controlling the particle size and morphology during the synthesis process [1,37]. This is an important advantage, as several studies have noted that particle agglomeration and the production of particles with a non-uniform size and shape tend to be some of the most prominent issues associated with the currently available synthesis techniques [37]. Other compounds such as polyethylene glycol (PEG) have also been used for the shape-controlled synthesis of ZnO nanoparticles, and it has been observed that varying the concentration of PEG can modify the morphology and size of the resulting particles [2,10]. However, the use of natural secretory carbohydrates from *C. vulgaris* for the shape-controlled synthesis of ZnO nanoparticles is likely to be more beneficial, as it provides an opportunity to utilise the waste material generated from the production of *C. vulgaris* biomass. Therefore, the aim of this study was to produce ZnO nanoparticles using secretory compounds (carbohydrates) from *C. vulgaris* via a controlled synthesis approach. This approach is likely to result in distinct, non-aggregated particles, and presents a more cost-effective and sustainable alternative to the existing production techniques.

## 2. Materials and Methods 

### 2.1. Chemicals

Zinc acetate dihydrate (Zn(OAc)_2_·2H_2_O, ACS grade) was purchased from Merck Millipore (Darmstadt, Germany) and ammonium hydroxide (NH_4_OH, 25%) was purchased from Sigma-Aldrich (St. Louis, MO, USA). The water used for media preparation and chemical reactions was Millipore water (Millipore Corp., Bedford, MA, USA, conductivity range = 0.055−0.294 l S/cm). BG -11 broth (M1541) was purchased from Himedia (Himedia Laboratories, Mumbai, India). The illumination was provided by cool white fluorescent lamps (20W FL T10 230V G13) from Pars Shahab Lamp Co. (Tehran, Tehran Province, Iran).

### 2.2. C. Vulgaris Culture Conditions

The *C. vulgaris* culture supernatant was prepared based on our previous reports [37]. During this process, the *C. vulgaris* cells (10^7^ cells/mL) were inoculated in a BG-11 broth medium, which is a universal medium for the cultivation and maintenance of blue cyanobacteria and freshwater algae, and incubated at 28 °C in a 16 h light/8 h dark cycle. At the end of the logarithmic growth phase (after 20 days of incubation), the culture was centrifuged (4000 *g*, 20 min), and the supernatant was harvested. The resulting clear and colourless solution was utilised for the synthesis of the ZnO nanorods.

### 2.3. Synthesis of the ZnO Nanorods 

The ZnO nanorods were synthesised at room temperature via a controlled reaction. As part of this process, 1 g of Zn(OAc)_2_·2H_2_O was dissolved in 140 mL of the *C. vulgaris* culture supernatant. Subsequently, 1.5 mL of ammonium hydroxide (NH_4_OH, 25%) was added dropwise to the mixture, and the solution was then kept under reflux at 80 °C. After 6 h, the product was washed with deionised water and further refluxed for another 9 h. Finally, the fabricated particles were washed several times with deionised water and dried at 60 °C in an oven for 24 h.

### 2.4. Analytical Methods

The morphology and size of the fabricated ZnO nanorods were characterised using transmission electron microscopy (TEM) (Philips, Eindhoven, Netherlands, CM10, HT 100 KV). Fourier transform infrared (FTIR) spectroscopy (PerkinElmer Spectrum One, Waltham, MA, USA) in the range of 400–4000 cm^−1^ was used for the verification of the ZnO nanorods. The crystal structure of the sample was evaluated by a Siemens D5000 diffractometer (Siemens, Germany, CuKα radiation, 10° ≤ 2Θ ≤ 80°). A UV-Vis absorption spectrum was also recorded in the range of 200-800 nm using a Chrom Tech (CT-8200) double-beam spectrometer (Chrom Tech, Inc. Singapore 608780). 

### 2.5. Antimicrobial Activity

The antimicrobial activity of the prepared nanorods was evaluated using the microdilution technique, which was developed according to the guidelines of the Clinical and Laboratory Standards Institute (CLSI) [38].

## 3. Results and Discussion

### 3.1. Characterisation of the ZnO Nanorods

#### 3.1.1. FTIR Spectra Analysis 

**Figure 1:**
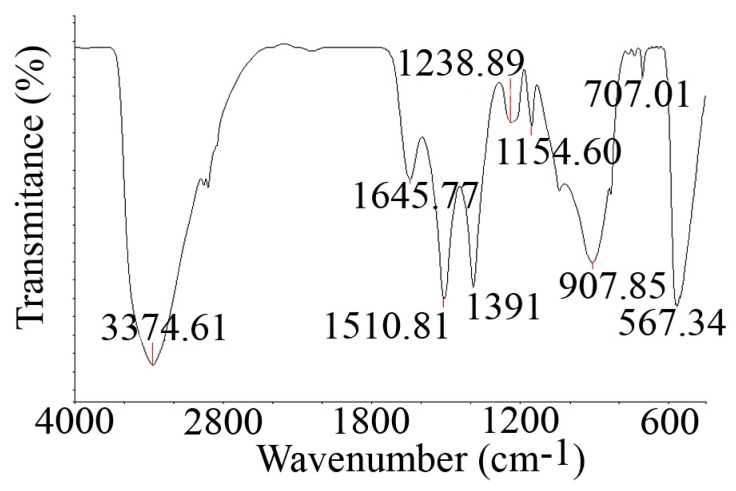
Fourier transform infrared (FTIR) spectrum of the zinc oxide (ZnO) nanorods.

The FTIR spectrum of the synthesised ZnO nanorods is provided in Figure 1. The absorption peak at 3374.61 cm^−1^ can be assigned to the stretching vibrations of the OH groups [5]. The band located at 1645.77 cm^−1^ can be correlated with the stretching vibration of the carbonyl groups. The peak at 1238.89 cm^−1^ can be attributed to the C–C bond [36]. The two intense peaks at 1510.81 cm^−1^ and 1391 cm^−1^ are indicative of nitro groups. The peak located at 567.34 cm^−1^ is the characteristic peak for ZnO, which corresponds to the stretching mode of the Zn–O bond [5].

#### 3.1.2. X-ray powder diffraction (XRD) Analysis 

**Figure 2:**
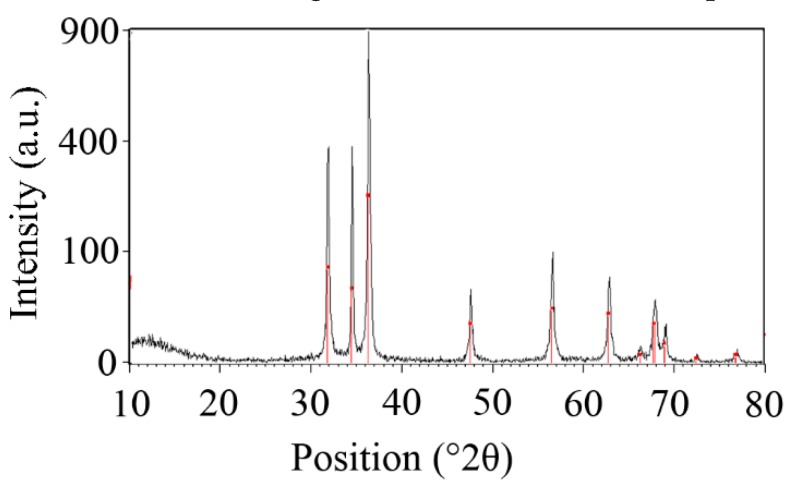
X-ray powder diffraction (XRD) pattern of the ZnO nanorods.

The X-ray diffraction pattern of the prepared nanorods is illustrated in Figure 2. The characteristic diffraction peaks observed at 31.9°, 34.4°, 36.2°, 47.5°, 56.5°, 62.8°, 66.1°, 67.9°, and 69° are in agreement with previous studies that have focused on the development of ZnO nanostructures [2,5,8,10,20,39,40,41]. The crystallite size of the manufactured nanorods was estimated to be 3.4 nm using the Scherrer calculator from X’Pert HighScore version 3.0.5 (Philips, Eindhoven, Netherlands).

#### 3.1.3. TEM Analysis 

**Figure 3:**
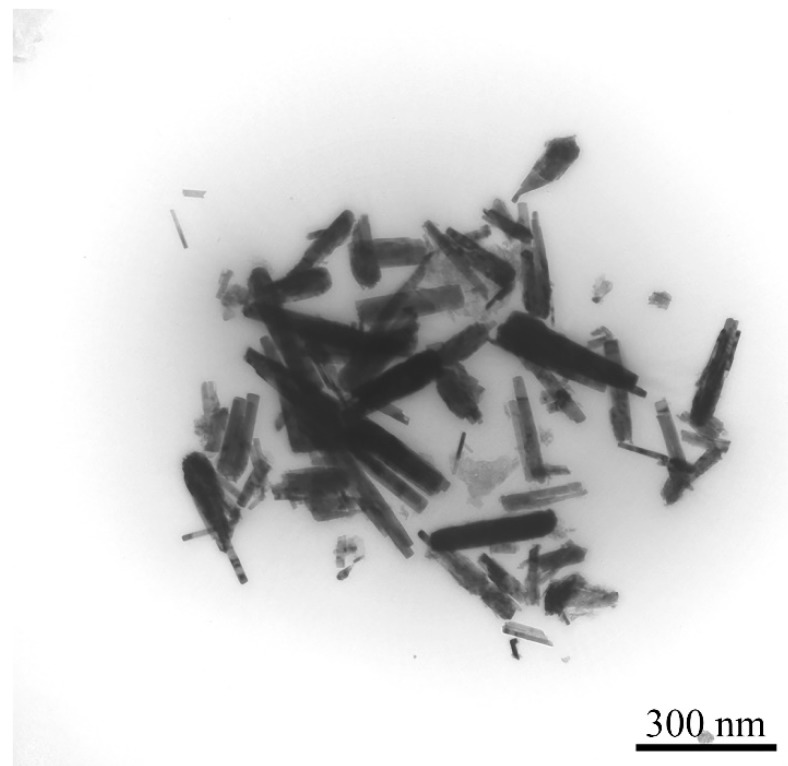
Transmission electron microscopy (TEM) photograph of the prepared ZnO nanorods.

**Figure 4:**
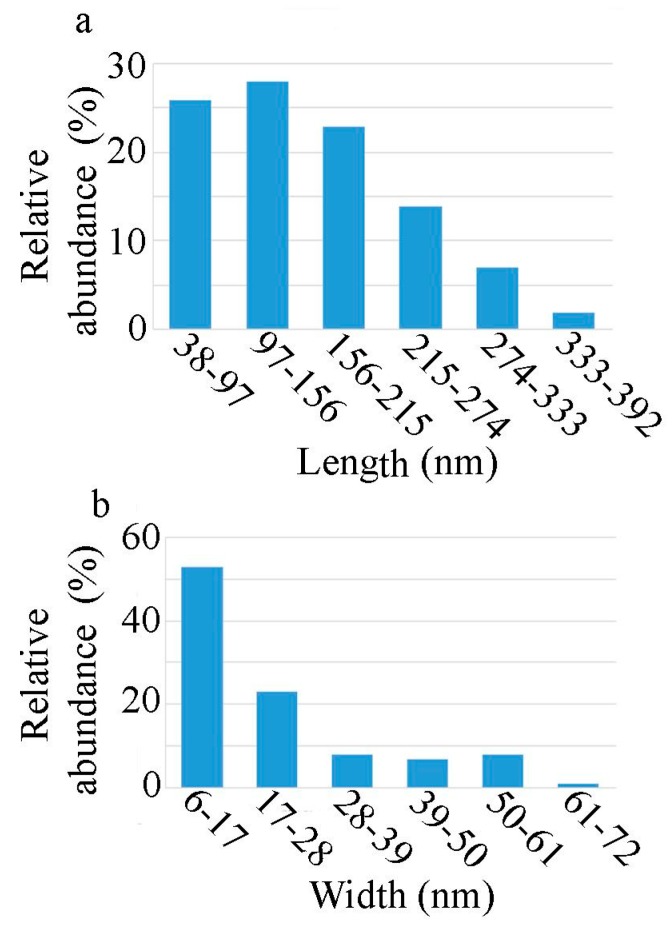
Histograms of the length (**a**) and the width (**b**) of the obtained ZnO nanorods.

The size distribution and morphology of the ZnO nanorods were evaluated using TEM analysis (Figure 3). The synthesised nanostructures had a rod-like shape, with an average length of 150 nm (Figure 4a) and an average width of 21 nm (Figure 4b). The aspect ratio (defined as the ratio of the length to the width) was calculated to be 7.14. The prepared particles were not uniform in shape and size and it can also be observed that, while the variation in the length of the nanorods is approximately normally distributed, the width is not (the distribution is slightly skewed to the right).

This study employed secretory compounds from *C. vulgaris* for the bio-assisted synthesis of ZnO nanorods. It can be observed from the TEM photograph (Figure 3) that the resulting ZnO nanorods were distinctly separate particles. This was achieved through the use of secretory compounds from *C. vulgaris*, which play an important role in controlling the growth pattern of the ZnO nanocrystals [37]. Therefore, the nature of the synthesised ZnO nanorods illustrates the success of the implemented approach. These results are in close agreement with previous reports that illustrate the shape-controlling role of biological compounds from *C. vulgaris*. In this regard, biomolecules from *C. vulgaris* can be grouped into two separate categories, namely cell extract compounds and secretory compounds. Each of these compounds can provide different nanostructures with different characteristics. Previous investigations indicated that proteins are the active compound in the *C. vulgaris* cell extract that is responsible in the bio-assisted synthesis of metal (particularly silver) nanoparticles [42]. Xie et al. showed that certain functional groups in the protein residues have a primary role in the fabrication of metal nanostructures [42]. In particular, they found that the hydroxyl groups present in tyrosine residues were the sites for metal ion reduction, and the carboxyl groups in the aspartic acid and glutamic acid residues play a shape-controlling role. These acidic residues facilitate the anisotropic growth of nanocrystals and are responsible for the formation of silver nanoplates [42]. Zhang et al. reported identical results in the bio-assisted synthesis of ZnO nanoparticles using *C. vulgaris* cell extract [43]. They found that using *C. vulgaris* extract as an additive in the synthesis reaction resulted in the formation of plate-like nanostructures. On the other hand, investigations focusing on the secretory compound of *C. vulgaris* interestingly indicated that carbohydrates are the effective compound in the formation of silver nanoparticles. The prepared nanostructures were uniform spherical particles that represented an isotropic growth pattern [36]. Similar results were also reported in the controlled synthesis of FeOOH nanospheres using secretory compounds from *C. vulgaris* [37]. However, the results from the current experiment revealed that secretory compounds from *C. vulgaris* do not always drive the isotropic growth of metal nanocrystals. The shape-controlling role of carbohydrates polymers in the growth of ZnO nanocrystals was also reported elsewhere [44]. Particularly, xanthan gum and PEG were reported as efficient shape-controlling agents [2,5,34]. It has been shown that by increasing the PEG concentration, the transformation of ZnO nanoparticles into rod-like structures occurred [2,34]. Hence, without any controlling agent, ZnO nanoparticles were formed [2,34,45].

#### 3.1.4. UV-Vis Spectra 

**Figure 5:**
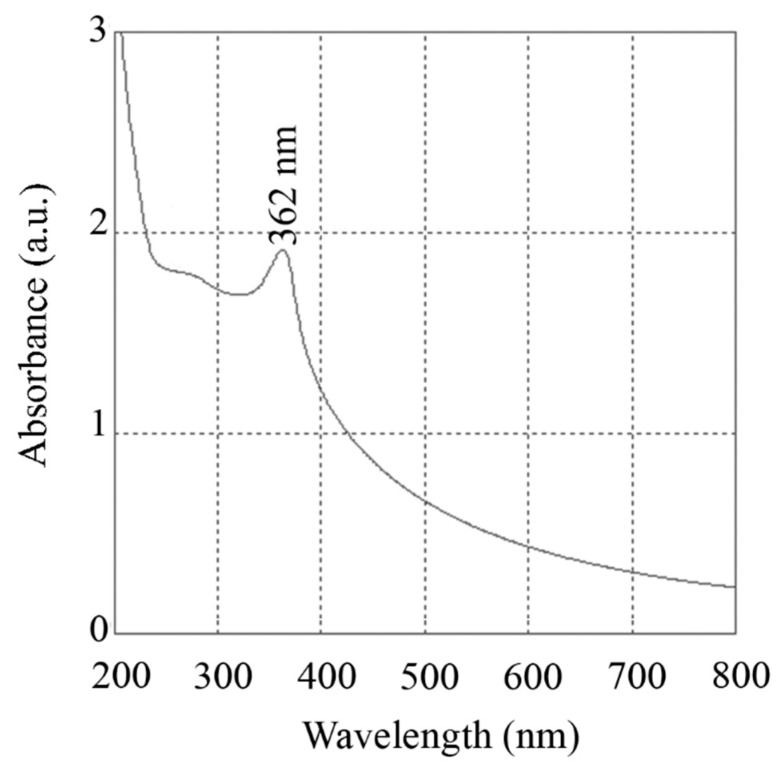
UV-Vis spectrum of the prepared ZnO nanorods, exhibiting an absorption peak at 362 nm.

The potential for the prepared ZnO nanorods to absorb UV-Vis irradiation was investigated, and the spectrum is depicted in Figure 5. The prepared nanorods exhibited an absorption peak at 362 nm, which exemplifies the characteristic absorption behaviour of ZnO nanostructures. This key property is a unique characteristic of ZnO particles, which makes them suitable for the production of valuable pharmaceutical compounds such as sunscreens. ZnO particles as an efficient sun blocker can provide protection against the adverse effects of UV light. ZnO particles are effective against UVA radiation, and broadband UV protection is commonly achieved by a combination of ZnO with particles of titanium dioxide (TiO_2_) [46]. Furthermore, the shoulder in the UV-Vis spectrum proves that the prepared nanorods are effective against both UVB (290–320 nm) and UVA (320–400 nm) radiations. The UV illumination effect also influences the biological activity of ZnO nanostructures [3]. ZnO possesses high photocatalytic efficiency, and its ability to absorb UV light significantly enhances its interaction with bacterial cells, facilitating growth inhibition or cell death through the generation of reactive oxygen species (ROS) [3].

### 3.2. Antimicrobial Activity

**Figure 6:**
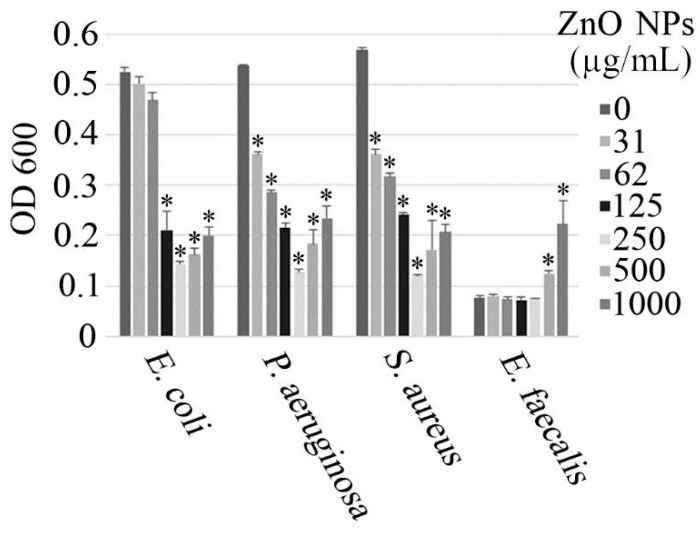
Antimicrobial activity of the ZnO nanorods against various Gram-positive and Gram-negative bacterial strains. OD, optical density; ZnO NPs, zinc oxide nanoparticles.

The antimicrobial activity of the ZnO nanorods was investigated against both Gram-positive (*Staphylococcus aureus* and *Enterococcus faecalis*) and Gram-negative (*Escherichia coli* and *Pseudomonas aeruginosa*) bacterial strains, and the results are illustrated in Figure 6. In all of the tested strains, except for *E. faecalis,* the antimicrobial activity of the ZnO nanorods was concentration-dependent, up to a concentration of approximately 250 µg/mL. At higher concentrations, a gradual reduction in the antimicrobial activity was observed, which may be due to the aggregation of the nanorods. Interestingly, high concentrations of the nanorods seem to promote the growth of *E. faecalis*, which appears to show resistance to the ZnO nanorods.

These observations are in contrast to those from various other studies, in which it has been noted that an increase in the nanoparticle concentration correlates with an increase in the antimicrobial activity [3,47,48,49,50]. Increasing the concentration of the nanorods may result in aggregation, which could alter both the morphology and the size of the resulting aggregates, in comparison to the individual particles. The aggregates are likely to have different shapes and a larger size relative to the discrete particles. This may reduce their antibacterial properties, as it has been observed that the antimicrobial properties depend on both the shape and size of the particles. Certain shapes sustain greater antimicrobial activity and larger particles, particularly in the micro size range, are not as potent as their nano-sized counterparts [3,5]. Furthermore, the increase in the nanoparticle concentration could potentially lead to a saturation effect, which could be another explanation for the observed behaviour. The inhibitory effects of the nanorods were enhanced as their concentration was raised to 250 μg/mL, at which point the maximum antimicrobial activity was observed. Consequently, above this concentration, any further increase in the nanoparticle concentration did not enhance the antimicrobial effects.

The trend observed in the antimicrobial activity of the ZnO nanorods for *E. faecalis* is considerably different to that observed for the other three bacterial strains that were investigated. The optical density (OD) at 600 nm was fairly consistent for a nanoparticle concentration from 0 μg/mL to 250 μg/mL, which suggests that the nanoparticle concentration within this range has little or no effect on the cell viability. An increase in the OD, and hence the cell viability, was noted as the nanoparticle concentration exceeded 250 μg/mL, which was similar to that observed for the other investigated bacterial strains. However, the results obtained for *E. faecalis* also suggest that a nanoparticle concentration greater than 250 μg/mL promotes cell growth.

The ZnO nanorods also appear to have a similar impact on the cell viability for both the Gram-positive and Gram-negative bacterial strains, as the variation in the cell viability in response to the nanoparticle concentration is comparable for all of the investigated strains, except for *E. faecalis* (as alluded to previously). This observation is in contrast to the findings of other studies, which suggest that Gram-positive bacteria are more susceptible to the antibacterial activity of ZnO nanoparticles, in comparison to Gram-negative bacteria, as a result of the differences in their cell wall structure, cell physiology, metabolism, and degree of contact [48,51]. However, these studies measured the zone of inhibition to quantify the antimicrobial activity, whereas the present study used absorbance measurements to assess the antibacterial activity. Hence, the different approaches used to evaluate the antimicrobial activity of the ZnO nanostructures may have an impact on the results obtained. Furthermore, those studies only compared the antimicrobial effects of ZnO nanostructures on *S. aureus* and *E. coli*, as opposed to the present study, which considered four different species. Thus, it is possible for the response of different Gram-positive and Gram-negative bacterial strains to vary. If only the *S. aureus* and *E. coli* strains are considered from the present study, then the results are comparable to those of previous investigations, as *S. aureus* is more sensitive to the nanorods in comparison to *E. coli*. 

It has been determined that the antimicrobial properties of metallic nanoparticles are attributable to their physicochemical properties [5,52,53]. The antibacterial activity of ZnO nanostructures varies considerably in the literature, and while numerous studies [39,41,47,48,50,53,54,55,56] have demonstrated that ZnO nanostructures have an adverse effect on microbial growth and metabolism, the study conducted by Ebrahiminezhad et al., has reported the synthesis of biocompatible xanthan gum-coated microstars, which exhibited no antimicrobial activity [5]. The variation in the antimicrobial activity of ZnO particles is largely due to the particle size, and it has been established that reduction of the particle size from the micro to the nano range results in nanoparticles that demonstrate potent antibacterial activity [3,5]. Furthermore, the use of biocompatible and other coatings is also likely to have an impact on the antimicrobial properties of the resulting particles [5]. 

Several means have been proposed for the antimicrobial activity of ZnO nanostructures, as the particles can interact with either the surface or the core of bacterial cells to induce various antibacterial mechanisms [3]. The three most probable mechanisms of the antibacterial activity include the generation of ROS, the release of zinc ions (Zn^2+^), and changes in the permeability of the bacterial cell membrane [3,4,56]. The generation of ROS is often considered to be the primary mechanism responsible for the antibacterial activity of ZnO nanostructures [3]. ROS target and destroy important cellular components such as DNA, phospholipids, and proteins, which results in either growth inhibition or cell death [3,50]. The release of Zn^2+^ into the surrounding medium is another prominent source of the antibacterial activity of ZnO nanostructures, as the released Zn^2+^ can bind to biomolecules such as proteins and carbohydrates, disrupt enzyme systems, and inhibit active transport and amino acid metabolism in bacterial cells, which has a detrimental effect on cell viability [3,50]. The penetration and accumulation of nanostructures in the cell membrane is another potential source of toxicity, as this results in the dissipation of the proton motive force and causes changes in the permeability of the plasma membrane, which results in the progressive release of lipopolysaccharides, membrane proteins, and intracellular factors from the bacterial cell, reducing cell viability [3].

## 4. Conclusions

An efficient method for the synthesis of ZnO nanorods was developed using secretory compounds from *C. vulgaris*. FTIR spectroscopy, XRD analysis, UV-Vis spectroscopy, and TEM were employed to characterise the synthesised nanostructures. The prepared particles were 150 nm and 21 nm in average length and width, respectively. Furthermore, the synthesised nanorods were able to block irradiations in both the UVB and UVA ranges. This unique property makes the particles a promising material for the fabrication of sunscreens with a broadband protection. The antimicrobial activity of the ZnO nanorods against both Gram-positive and Gram-negative bacterial strains was also assessed using the micro-dilution method. It was observed that the ZnO nanorods demonstrated notable antibacterial activity, and a concentration of 250 µg/mL was determined to be the most effective. The antibacterial properties of the synthesised ZnO nanorods enable it to be employed in food packaging and in the medical field, as well as a range of other antimicrobial applications. It also is worthwhile to mention that secretory compounds from *C. vulgaris* are available as culture supernatant, which is a by-product of microalgae biomass-producing plants. The use of this inexpensive material can reduce the costs associated with the process and can provide a more economical alternative to the chemicals that are commonly employed in the synthesis of nanostructures. Additionally, it is obvious that the concentration of controlling agents has a significant impact on the prepared nanostructures. Therefore, by employing diluted culture supernatant, nanostructures with different properties may be obtained. This aspect presents an interesting research opportunity that can be investigated in future experiments.
